# As Consequências Não Aparentes Fora do Espectro da COVID da Pandemia de COVID-19

**DOI:** 10.36660/abc.20200935

**Published:** 2020-11-01

**Affiliations:** 1 Escola Bahiana de Medicina SalvadorBA Brasil Escola Bahiana de Medicina, Salvador, BA - Brasil; 2 Hospital Santa Izabel da Santa Casa da Bahia SalvadorBA Brasil Hospital Santa Izabel da Santa Casa da Bahia, Salvador, BA – Brasil

**Keywords:** Pandemia, COVID-19, Betacoronavírus, Cardiologia, Oncologia, Hospitalização, Serviços Médicos de Emergência

O mundo tem enfrentado uma pandemia de COVID-19 de enormes proporções. A maioria dos lugares estava despreparada para lidar com essa situação^1^ e considerando sua ocorrência no atual estágio de informações rápidas, generalizadas e ocasionalmente não filtradas, o aumento considerável do medo afetou a maioria das pessoas no mundo. Principalmente com a rápida contaminação de pessoas nos hospitais e nas comunidades, conforme anunciado no noticiário, e potencializado por fotos de pacientes esperando, desassistidos, fora dos centros médicos, por falta de suporte adequado.

Este cenário apocalíptico foi visto em muitos lugares considerados como adequadamente equipados para o atendimento regular dos problemas de saúde do dia a dia. Todas as atenções foram desviadas para fornecer instalações e equipamentos para o atendimento dos pacientes afetados pela COVID-19 e seus familiares.^2^

Entretanto, um problema invisível estava surgindo no meio de tudo isso. O atendimento aos pacientes precisando de cuidados devido a outras doenças que não COVID, mas ainda assim graves.

Almeida et al.^3^ nesta edição dos Arquivos Brasileiros de Cardiologia, demonstraram uma visão acurada para o problema quando, em sua cidade de Feira de Santana na Bahia-Brasil, foram um dos primeiros a documentar tal problema analisando a redução considerável no atendimento a doenças cardíacas, oncológicas e outras condições potencialmente incapacitantes. Outros têm identificado esse problema em muitas partes do mundo, com graves consequências de morte súbita fora do hospital, infarto do miocárdio (IM) não tratado, chegada tardia ao hospital por IM e, como consequência, ruptura ventricular, como há muito tempo não se via, choque cardiogênico, insuficiência cardíaca^4^ cirurgia cardíaca,^5^^,^^6^ entre outras consequências, sem contar a perda de oportunidade do diagnóstico precoce do câncer, bem como quimioterapia e radioterapia adequadas. Apendicite supurativa, úlceras gástricas perfuradas também foram documentadas.

À medida que as instalações hospitalares iam adquirindo progressivamente o suporte necessário e a transmissão se estabilizava e, em muitos casos, diminuía, a conscientização trazida por observações como a relatada neste número dos Arquivos Brasileiros de Cardiologia suscitou atitudes imediatas para facilitar a captação de casos não-COVID que necessitam dessa atenção.^7^^,^^8^

Essa nem sempre é uma tarefa simples^9^ e a proteção dos pacientes e seus familiares deve ser garantida por meio de questionários, exames para COVID em pacientes, familiares e pessoal médico, higienização adequada do ambiente e, sempre que possível, com fluxos distintos para esses pacientes.

Até que a muito esperada chegada de uma vacina eficaz aconteça, deve-se lembrar que a humanidade tem esperanças de uma situação pós-pandêmica, mas deve ser lembrada que em termos populacionais não haverá um Pós-COVID 19. Essa ameaça nos acompanhará por muitos anos e, portanto, sempre haverá a necessidade de precauções de contaminação e adoção de melhores condições de saneamento.
