# Supplementary material for: As Consequências Não Aparentes Fora do Espectro da COVID da Pandemia de COVID-19
Source: Arq Bras Cardiol. 2020 Nov 1;115(5):871–2. [Article in Portuguese] doi: 10.36660/abc.20200935 (PMC8452197; doi:10.36660/abc.20200935)
Supplement: Translation

# Translation: The Unapparent Non-COVID Consequences of the COVID-19 Pandemic

**Affiliations:** 1 SalvadorBA Brazil Escola Bahiana de Medicina, Salvador, BA - Brazil; 2 SalvadorBA Brazil Hospital Santa Izabel da Santa Casa da Bahia, Salvador, BA – Brazil

**Keywords:** Pandemics, COVID-19, Betacoronavirus, Cardiology, Oncology, Hospitalization, Emergency Medical Services

The world has been facing a COVID-19 pandemics of enormous proportion. Most places were unprepared to cope with this situation^1^ and considering its occurrence in the current scenario of fast, widespread and occasionally unfiltered information, the considerable growth of fear has affected most people worldwide. Mainly considering the rapid contamination of people in the hospitals and in the communities, as announced by the news, and potentialized by pictures of unassisted patients waiting outside the medical facilities, due to the lack of adequate support.

This apocalyptic scenario was seen in many places that were considered as adequately equipped for the regular treatment of ordinary health problems. All attention was diverted to provide installations and equipment to take care of the COVID-19 affected patients and their relatives.^2^

However, an unseen problem was emerging in the middle of all this. The care for the patients in need of attention for non-COVID and yet, severe conditions.

Almeida et al.,^3^ in this issue of *Arquivos Brasileiros de Cardiologia*, demonstrate an accurate view of the problem, as they were among the first to document such a problem in their municipality of Feira de Santana in Bahia, Brazil, by analyzing a considerable reduction in the treatment of heart disease, oncologic disease and other potentially incapacitating conditions. Others have identified such problems in many parts of the world, with severe consequences of out-of-the-hospital sudden death, untreated myocardial infarction (MI), late hospital arrival for MI and as a consequence, ventricular rupture, as it had not been seen for a long time, and cardiogenic shock, heart failure^4^ cardiac surgery,^5^^,^^6^ and other consequences, not mentioning the loss of opportunity for early diagnosis of cancer, as well as appropriate chemotherapy and radiotherapy. Suppurative appendicitis and perforated gastric ulcers have also been documented.

As the hospital facilities progressively acquired the necessary support and the transmission stabilized, and in many cases declined, the awareness brought on by observations such as the one reported in this issue of the *Arquivos Brasileiros de Cardiologia* prompted immediate attitudes toward facilitating the treatment of non-COVID cases in need of such care.^7^^,^^8^

This is not always a simple task^9^ and the protection of the patients and their relatives have to be guaranteed by means of questionnaires, COVID testing for the patients, relatives and medical personnel, adequate hygienization of the environment and whenever possible, with distinct flows for these patients.

Until the arrival of a much expected effective vaccine, one must remember that humanity hopes for a post-pandemic situation but must be reminded that, in populational terms, there will not be a Post-COVID 19 era. This threat will accompany us for many years and, therefore, there will always be a need for precautions against contamination and the adoption of better sanitation conditions.
